# Efficacy and tolerability of generic pirfenidone after switch from Esbriet^®^ in idiopathic pulmonary fibrosis: a real-world observational study

**DOI:** 10.1007/s11739-026-04343-9

**Published:** 2026-04-17

**Authors:** Roberto Tonelli, Maria Giulia Turchiano, Antonio Moretti, Dario Andrisani, Filippo Gozzi, Giulia Raineri, Anna Valeria Samarelli, Federica Andolfi, Valentina Ruggieri, Enrico Clini, Stefania Cerri

**Affiliations:** 1 Respiratory Disease Unit, University Hospital of Modena, Modena, Italy; 2 Respiratory Disease Unit, Santa Maria Bianca Hospital, Mirandola, Italy; 3 Center for Rare Lung Diseases, University Hospital of Modena, Modena, Italy; 4 Department of Surgical and Medical Science, Laboratory of Experimental Pneumology, University of Modena and Reggio Emilia, Modena, Italy; 5 Research Office, University Hospital of Modena, Modena, Italy

**Keywords:** Idiopatic pulmonary fibrosis, Pirfenidone, Drug therapy

## Abstract

**Background:**

Generic formulations of pirfenidone are increasingly adopted in idiopathic pulmonary fibrosis (IPF), yet real-world evidence supporting their clinical equivalence to the originator remains limited. We aimed to evaluate whether switching from branded pirfenidone (Esbriet^®^) to a generic formulation affects treatment efficacy or tolerability.

**Methods:**

We conducted a retrospective, within-patient observational study including consecutive patients with IPF treated with Esbriet^®^ for ≥ 6 months before switching to generic pirfenidone. Pulmonary function was assessed at three time points: 6 months before the switch (T − 6), at switch (T0), and 6 months after (T + 6). The primary endpoint was the within-patient percentage change in FVC over two consecutive 6-month periods (T − 6 → T0 vs T0 → T + 6), analysed within a pre-specified equivalence framework (± 5 percentage points). Secondary endpoints included DLCO changes and treatment-related adverse events (AEs), analysed at the patient level using paired comparisons.

**Results:**

Sixty-five patients (median age 77.0 years [72.3–80.0] years, 78% male) had complete functional follow-up. The mean percentage decline in FVC was − 1.9% before the switch and − 1.7% after the switch. The estimated between-period difference in FVC change was 0.2 percentage points (95% CI − 1.1 to 1.5), fully contained within the pre-specified equivalence margins. Similar findings were observed for DLCO, with no significant difference between periods. Overall, 43% of patients experienced at least one AE during treatment. Gastrointestinal AEs were the most frequent, but paired analyses showed no significant difference in patient-level AE occurrence between branded and generic periods. No severe AEs or treatment discontinuations were observed.

**Conclusions:**

In this real-world cohort of patients with IPF, switching from branded to generic pirfenidone was not associated with clinically meaningful differences in lung function decline or treatment tolerability.

**Supplementary Information:**

The online version contains supplementary material available at 10.1007/s11739-026-04343-9.

## Introduction

Idiopathic pulmonary fibrosis (IPF) is a chronic, progressive interstitial lung disease associated with irreversible decline in lung function and poor prognosis [1]. Over the past decade, disease-modifying therapies have reshaped IPF management, with nintedanib and pirfenidone established as the main antifibrotic treatments shown to slow functional decline, and novel agents such as nerandomilast recently emerging as additional therapeutic options targeting complementary pathogenic pathways [2–4]. Despite these advances, IPF remains a fatal disease, and long-term treatment effectiveness relies not only on antifibrotic efficacy but also on tolerability and treatment persistence [5]

Pirfenidone was among the first antifibrotic agents to demonstrate clinical efficacy in IPF [6]. In the CAPACITY and ASCEND randomized controlled trials, pirfenidone administered at a target dose of 2403 mg/day significantly reduced the rate of forced vital capacity (FVC) decline compared with placebo, with a consistent safety profile in patients with mild-to-moderate IPF [3, 7]. Pooled analyses and long-term extension studies further supported sustained efficacy and acceptable tolerability in routine clinical practice [8].

Pirfenidone formulations have been approved on the basis of pharmacokinetic bioequivalence to the originator drug, including comparable C_max and AUC values in healthy volunteers [9–12]. Following patent expiration, several generic formulations became available. However, regulatory bioequivalence does not necessarily imply clinical equivalence in patients with IPF, who are typically older, multimorbid, and exposed to long-term treatment [13]. In real-world settings, pirfenidone-related adverse events (AEs)—most commonly gastrointestinal and skin-related—are frequent and may influence adherence, dose reductions, or treatment interruptions, factors that are known to affect long-term effectiveness [13–16]. Importantly, existing real-world evidence on pirfenidone largely reflects experience with the branded formulation, while data specifically addressing the clinical impact of switching to generic formulations in IPF are scarce.

To date, no randomized or observational head-to-head studies have directly compared branded and generic pirfenidone with respect to functional outcomes or tolerability in patients with IPF [17]. As a result, clinical interchangeability between formulations is largely inferred from pharmacokinetic equivalence rather than supported by direct clinical evidence.

This study aims at evaluating whether switching from branded pirfenidone (Esbriet^®^) to generic formulations is associated with clinically meaningful differences in lung function decline or treatment tolerability in a real-world cohort of patients with IPF, using a within-patient observational design.

## Materials and methods

### Study design and population

This retrospective study was conducted at the Centre for Rare Lung Diseases (MaRP), University Hospital of Modena, Italy, between January 2023 and June 2024. All consecutive patients with a confirmed diagnosis of IPF, according to ATS/ERS/JRS/ALAT guidelines 1, were screened.

Inclusion criteria were as follows: treatment with branded pirfenidone (Esbriet^®^) for at least 6 months; switch to a generic formulation (Sandoz, Teva, Zentiva, or Dr Reddy’s) administered at the same dosage (801 mg three times daily); and availability of pulmonary function data at 6 months before the switch (T–6), at the time of switch (T0), and 6 months thereafter (T + 6).

Patients experiencing acute exacerbation or prolonged treatment interruption (> 2 weeks, to avoid potential confounding related to treatment non-persistence) during the observation period were excluded. The transition to generic pirfenidone occurred as part of a hospital pharmacy policy rather than physician decision, thereby reducing the risk of selection bias. This study was approved by the local Ethics Committee (protocol number 432/2024, Area Vasta Emilia Nord) and conducted in accordance with the Declaration of Helsinki. Written informed consent for the use of clinical data for research and publication purposes was obtained from patients whenever available, in accordance with local regulations for retrospective studies.

### Data collection and outcomes definition

Demographic and clinical data were extracted from electronic medical records. Spirometry and DLCO were performed in the same laboratory by certified technicians in accordance with ERS/ATS standards.

The primary endpoint was the within-patient percentage change in forced vital capacity (FVC) over two consecutive 6-month periods, comparing the 6 months preceding the switch (T − 6 to T0; branded pirfenidone) with the 6 months following the switch (T0 to T + 6; generic pirfenidone). Percentage FVC change was calculated as follows: [(FVC at follow-up – FVC at baseline)/FVC at baseline] × 100. The primary analysis was conducted within an equivalence framework, under the hypothesis that switching to generic pirfenidone would not result in a clinically meaningful acceleration of FVC decline (see below). Secondary endpoints included the within-patient percentage change in DLCO over the same two 6-month periods and the occurrence of AEs before and after the switch. AEs were derived from routine clinical documentation and patient reports recorded during follow-up visits. No formal standardized reporting form was used in routine clinical practice; for study purposes, adverse events were retrospectively grouped into predefined clinically relevant categories (gastrointestinal, skin related, and other).

### Statistical analysis

Continuous variables are reported as median values with interquartile range (IQR), while categorical variables are expressed as counts and percentages.

Comparison between within-patient change in FVC and DLCO was performed using a linear mixed-effects model to account for repeated measures within individuals. The model included time, treatment period, and their interaction (time × treatment period) as fixed effects, with a random intercept for each patient. The effect of interest was the time × treatment period interaction, reported as an estimated mean difference with 95% confidence intervals (CI). The primary inference was performed within an equivalence framework using a pre-specified equivalence margin as described before; equivalence was concluded if the 95% CI for the between-period difference lay entirely within the equivalence limits. A pre-specified equivalence margin of ± 5 percentage points in FVC% and DLCO% decline over 6 months was defined a priori. This threshold was selected as a clinically anchored and pragmatic margin, rather than as a direct estimate of the minimal clinically important difference, based on evidence that declines of ≥ 5% predicted over 6–12 months are associated with clinically relevant progression and adverse outcomes in IPF [18]. AE outcomes were analysed at the patient level using paired comparisons (McNemar test) between the branded and generic treatment periods. For each AE category, paired risk differences and corresponding 95% CI were estimated using the Newcombe method for paired binary data. Exploratory analyses were performed stratifying AEs by treatment period (branded vs generic pirfenidone) to evaluate whether the association between baseline comorbidities and category-specific AEs differed across treatments. As an exploratory analysis, a multivariable logistic regression model was also fitted to assess whether baseline functional severity was associated with the occurrence of any treatment-related adverse event during the observation period.

All statistical analyses were performed using SPSS Statistics (IBM Corp., Armonk, NY, USA). Graphical representations were generated using GraphPad Prism (GraphPad Software, San Diego, CA, USA).

## Results

### Baseline characteristics

**Fig. 1:**
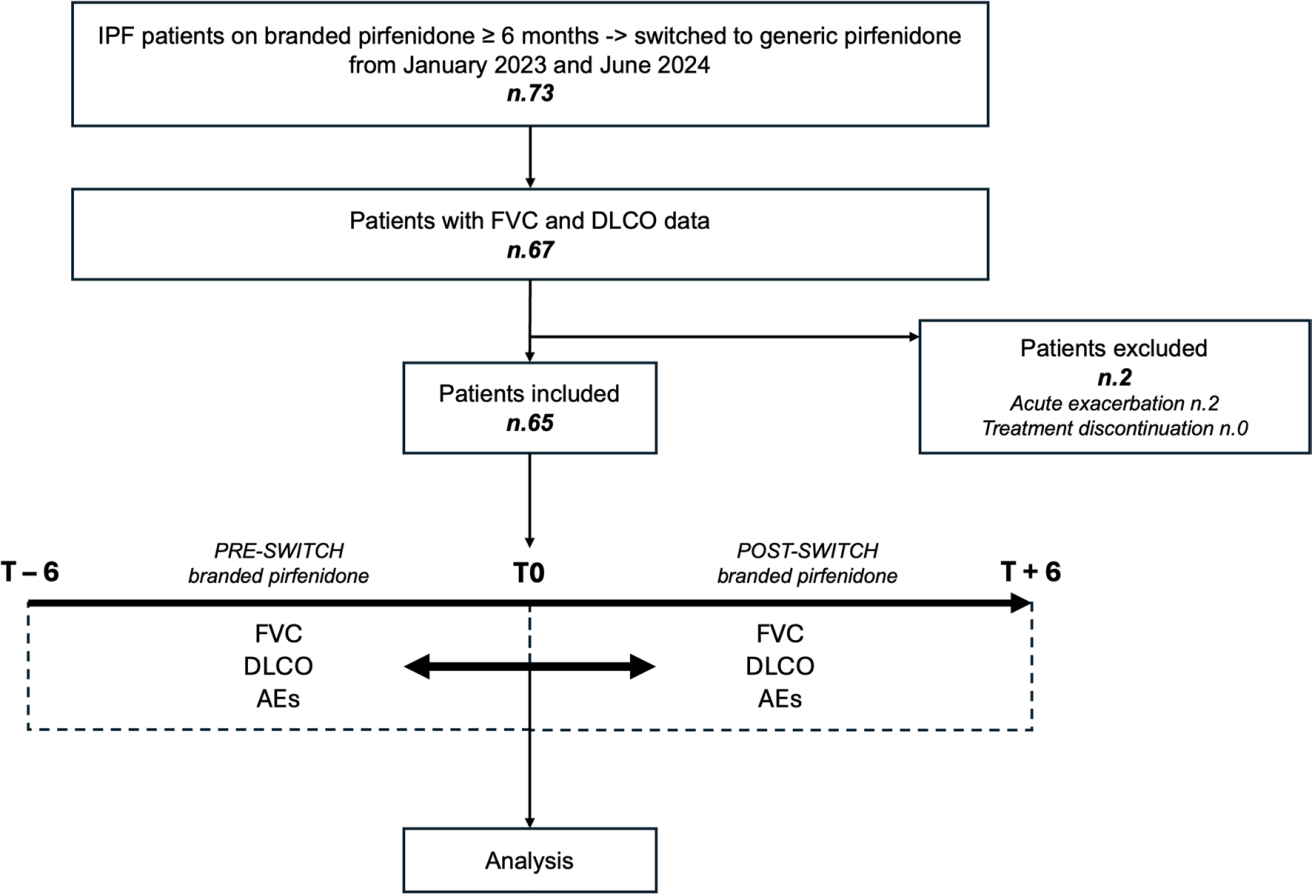
Study design and within-patient switch from branded to generic pirfenidone. Flowchart illustrating patient selection and the within-patient study design. Patients with IPF treated with branded pirfenidone for ≥ 6 months and switched to generic pirfenidone between January 2023 and June 2024 were screened (*n* = 73). Of these, 67 patients had complete functional data; two were excluded due to acute exacerbation occurring during the pre-switch period, resulting in 65 patients included in the analysis. Lung function parameters (FVC, DLCO) and AEs were assessed over two consecutive 6-month periods before (T − 6 to T0) and after (T0 to T + 6) the formulation switch, allowing paired within-patient comparison. *FVC* forced vital capacity*, DLCO* diffusing capacity of the lung for carbon monoxide*, IPF* idiopathic pulmonary fibrosis*, AE* adverse event

Seventy-three patients underwent switching, of whom sixty-seven had complete FVC and DLCO data. Two patients were excluded due to acute exacerbation during the pre-switch period, resulting in sixty-five patients included in the analysis. The study flowchart is shown in Fig. 1. Baseline demographic, clinical, and functional characteristics are summarized in Table 1. The study population reflected a typical real-world IPF cohort, with a median age of 77.0 years (IQR 72.3–80.0) and a marked male predominance (78%). Most patients had a history of smoking (91%). Cardiovascular comorbidities were highly prevalent (91%), while at least one additional non-cardiovascular comorbidity was present in 43% of patients. Additional respiratory diseases, mainly chronic obstructive pulmonary disease or bronchiectasis, were observed in 43% of the cohort. Gastrointestinal comorbidities were reported in 51%, skin disorders in 9%, and a history of malignancy in 22%. Fourteen patients (21%) were receiving long-term oxygen therapy at baseline.

**Table 1:** Demographic, clinical, and functional characteristics of the study population

Variable	Overall population (*n* = 65)
*Demographic characteristics*	
Age, years (IQR)	77.0 (72.3–80.0)
Male sex, *n* (%)	51 (78%)
Smoking history, *n* (%)	60 (91%)
*Clinical characteristics*	
Diagnosis	
Based on HRCT only, *n* (%)	43 (66%)
Based on biopsy, *n* (%)	22 (34%)
Supplemental oxygen, *n* (%)	14 (21%)
At least one comorbidity, *n* (%)	28 (43%)
Pulmonary, *n* (%)	28 (43%)
Cardiovascular, *n* (%)	59 (91%)
Gastrointestinal, *n* (%)	33 (51%)
Skin, *n* (%)	6 (9%)
Tumour history, *n* (%)	14 (22%)
Previous nintedanib exposure, *n* (%)	5 (8%)
*Pulmonary function test*	
T–6 FVC, %pred (IQR)	93.0 (79.5–104.8)
T–6 DLCO, %pred (IQR)	54.1 (48.0–64.8)
T0 FVC, %pred (IQR)	90.5 (78.5–104.0)
T0 DLCO, %pred (IQR)	49.0 (42.6–60.8)
T + 6 FVC, %pred (IQR)	89.0 (73.3–104.0)
T + 6 DLCO, %pred (IQR)	47.5 (35.3–58.5)

Values are reported as median (interquartile range, IQR) for continuous variables and as number (percentage) for categorical variables. Pulmonary function parameters are expressed as percentage of predicted values and are reported at 6 months before the switch to generic pirfenidone (T–6), at the time of switch (T0), and 6 months after the switch (T + 6)

*DLCO* diffusing capacity of the lung for carbon monoxide*, FVC* forced vital capacity, *HRCT* high-resolution computed tomography*, IQR* interquartile range*, %pred* percentage of predicted value

At T − 6, median FVC was 93.0% predicted (IQR 79.5–104.8), and median DLCO was 54.1% predicted (IQR 48.0–64.8). No treatment discontinuations lasting more than 14 days were observed across the two study periods.

### Functional outcomes

**Fig. 2:**
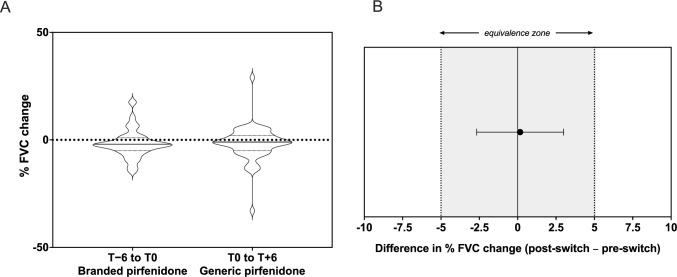
Percentage change in FVC before and after switching from branded to generic pirfenidone. Panel** A** shows the distribution of percentage FVC change during the 6 months before (T − 6 to T0) and after (T0 to T + 6) the switch. Panel** B** displays the model-based estimated difference in percentage FVC change between periods, with 95% CI. The shaded area represents the pre-specified equivalence zone (± 5 percentage points). The CI lies entirely within the equivalence margins, supporting clinical equivalence. *FVC* forced vital capacity*, CI* confidence interval

**Fig. 3:**
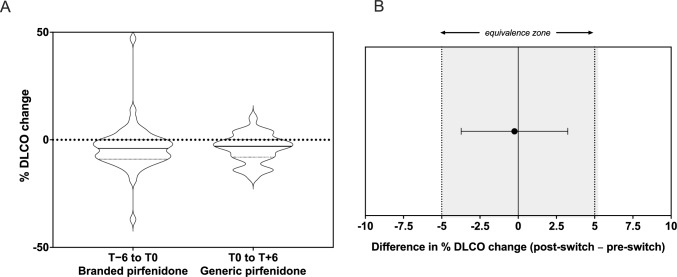
Percentage change in DLCO before and after switching from branded to generic pirfenidone. Panel **A** shows the distribution of percentage DLCO change during the 6 months before (T − 6 to T0) and after (T0 to T + 6) the switch. Panel **B** displays the model-based estimated difference in percentage DLCO change between periods, with 95% CI. The shaded area represents the pre-specified equivalence zone (± 5 percentage points). The CI lies entirely within the equivalence margins, supporting clinical equivalence. *DLCO* diffusing capacity of the lung for carbon monoxide*, CI* confidence interval

Over the 6 months preceding the switch (T − 6 to T0), the mean percentage change in FVC was − 1.9%, compared with − 1.7% over the 6 months following the switch to generic pirfenidone (T0 to T + 6). The distribution of percentage FVC change over the two periods is shown in Fig. 2 (panel A). In the linear mixed-effects model, the estimated between-period difference in percentage FVC change (post-switch minus pre-switch) was 0.2 percentage points (95%CI − 1.1 to 1.5) (Fig. 2**,** panel B). Across the two consecutive 6-month periods, the mean percentage change in DLCO was − 3.8% before the switch (T − 6 to T0) and − 4.0% after the switch to generic pirfenidone (T0 to T + 6) as shown in Fig. 3 (panel A). In the linear mixed-effects model, the estimated between-period difference in percentage DLCO change (post-switch minus pre-switch) was − 0.2 percentage points, with a 95%CI from − 1.9 to 1.5 percentage points Fig. 3**,** panel B. For both FVC and DLCO, the time × treatment period interaction was not statistically significant.

### Adverse events

**Fig. 4:**
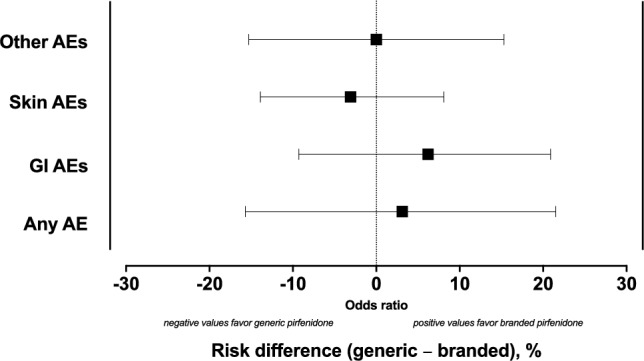
Patient-level risk differences for treatment-related adverse events between generic and branded pirfenidone. The figure shows the paired risk differences in the occurrence of treatment-related AEs at the patient level, comparing generic with branded pirfenidone. Risk differences and corresponding 95% CIs are reported for any AE, gastrointestinal AEs, skin-related AEs, and other AEs. Positive values indicate a higher risk during generic pirfenidone treatment, while negative values indicate a higher risk during branded pirfenidone treatment. Risk differences were estimated using the Newcombe method for paired binary data. *CI confidence interval, AE adverse event*, *FVC* forced vital capacity, *DLCO* diffusing capacity of the lung for carbon monoxide, *IPF* idiopathic pulmonary fibrosis, *AE* adverse event, *CI* confidence interval, *IQR* interquartile range, *OR* odds ratio, *LTOT* long-term oxygen therapy

Overall, 28 of 65 patients (43%) experienced at least one AE during treatment with pirfenidone; patient-level distributions of AEs across treatment periods are reported in Table 2, while the detailed event-level breakdown is provided in eTable 1 in the Supplementary Material. No severe AEs were reported. In one patient, a switch back to branded pirfenidone occurred due to a non-severe AE, without treatment discontinuation. Baseline FVC and DLCO were not significantly associated with the occurrence of any AE (OR 1.003, 95% CI 0.72–1.04; *p* = 0.87 and OR 1.02, 95% CI 0.98–1.07. *p* = 0.34, respectively). Gastrointestinal AEs were the most frequently reported (17 patients, 26%), followed by skin-related AEs (7 patients, 11%) and other AEs (15 patients, 23%). Paired risk differences (generic vs branded) for any AE, gastrointestinal, skin-related, and other AEs are shown in Fig. 4. The paired risk difference was 3.1% for any AE and 6.2% for gastrointestinal AEs, and − 3.1% and 0.0% for skin-related and other AEs, respectively. Baseline gastrointestinal comorbidities were not significantly associated with gastrointestinal AEs during either branded (OR 2.15, 95% CI 0.49–9.45; *p* = 0.30) or generic pirfenidone treatment (OR 2.24, 95% CI 0.60–8.35; *p* = 0.23), although effect estimates were directionally consistent across periods. Baseline skin comorbidities were associated with skin-related AEs during branded treatment (OR 9.33, 95% CI 1.19–72.99; *p* = 0.03), while no such events occurred during the generic period in this subgroup.

**Table 2:** Patient-level distribution of treatment-related adverse events

Adverse event category	Total (*n* = 65)	Branded pirfenidone	Generic pirfenidone	*p*-value	Both periods
Any adverse event	28 (43%)	11 (17%)	13 (20%)	0.84	4 (6%)
Gastrointestinal adverse events	17 (26%)	5 (8%)	9 (14%)	0.42	3 (4%)
Skin-related adverse events	7 (11%)	4 (6%)	2 (3%)	0.69	1 (1%)
Other adverse events	15 (23%)	7 (11%)	7 (11%)	0.99	1 (1%)

Adverse events are reported at the patient level and categorized according to their occurrence during the pre-switch period (T − 6 to T0) with branded pirfenidone, the post-switch period (T0 to T + 6) with generic pirfenidone, or both periods. Percentages are calculated using the total study population as the denominator (*n* = 65). Paired comparisons between periods were performed using McNemar’s test, accounting for within-patient changes across treatment periods

*AE* adverse event

## Discussion

This within-patient real-world study evaluated the clinical impact of switching from branded to generic pirfenidone in patients with IPF. Lung function decline, assessed by changes in FVC and DLCO, remained within the predefined equivalence margins between the pre- and post-switch periods. The overall incidence and distribution of treatment-related AEs were comparable between formulations. Exploratory analyses indicated that AEs tended to cluster in patients with specific comorbidity profiles, particularly gastrointestinal and cardiovascular comorbidities, independent of formulation.

### Functional outcomes

The magnitude of lung function decline observed both before and after the switch from branded to generic pirfenidone is fully consistent with that reported in large real-world cohorts, including the Italian IRENE study and other multicentre observational experiences, where mean FVC decline over 12 months typically ranges between 70 and 100 mL, with marked interindividual variability [14]. This confirms that the study population follows an expected real-world disease trajectory, providing a clinically appropriate framework for interpreting changes observed around formulation switching. Against this background, we focused on whether switching formulation was associated with a systematic deviation from the expected individual trajectory of lung function decline beyond clinically meaningful margins. Such an approach is particularly relevant in IPF, a disease characterized by non-linear progression and substantial within-patient variability, in which short-term changes in FVC may occur independently of treatment exposure [19, 20]. In this setting, the absence of any detectable divergence between pre- and post-switch trajectories suggests that formulation switching does not introduce a measurable perturbation of the disease course. Similar findings were observed for DLCO, with no evidence of divergence between pre- and post-switch trajectories. To our knowledge, this is the first study to specifically assess DLCO trajectories before and after switching between branded and generic pirfenidone. The equivalence margin of ± 5 percentage points in FVC (and DLCO) decline over 6 months reflects its established clinical and prognostic relevance in IPF, as an absolute decline of ≥ 5% predicted over 6–12 months is consistently associated with disease progression and increased mortality, and aligns with estimates of the minimal clinically important difference for FVC, typically ranging between 2 and 6% [1, 21]. This finding should be interpreted within the broader context of IPF drug development, where apparent signals observed in small or uncontrolled studies frequently attenuate when evaluated using more rigorous designs or larger datasets [22]. As recently discussed by Trachalaki et al*.*, this phenomenon—described as “regression to the truth”—highlights the risk of overinterpreting nominal differences in a disease with intrinsically noisy outcome measures 5. By anchoring the analysis to predefined margins of clinical relevance, the present study allows a clinically oriented evaluation of formulation switching. Importantly, these results are also coherent with post hoc analyses of pivotal pirfenidone trials showing that clinically meaningful declines in FVC do not necessarily indicate treatment failure, as continued therapy remains associated with a reduced risk of subsequent decline or death [8]. Although pretreatment rates of FVC decline may help identify subgroups with differential long-term benefit, individual responses remain heterogeneous and cannot be reliably inferred from short-term trajectory changes alone [19, 23, 24]. Taken together, these considerations support the clinical equivalence of branded and generic pirfenidone with respect to short-term functional outcomes, while reinforcing the need to interpret lung function trajectories in IPF within a framework that prioritizes clinical relevance over nominal differences.

### Adverse events

In the present cohort, the incidence and pattern of AE were comparable before and after the switch from branded to generic pirfenidone, with no signal suggesting a formulation-specific effect on tolerability. This finding is consistent with the well-established safety profile of pirfenidone, in which gastrointestinal and skin-related events represent the most frequent adverse reactions across both clinical trials and real-world studies [25]. Further, baseline functional severity was not significantly associated with the occurrence of AE, although this finding should be interpreted cautiously given the limited sample size and number of events. Beyond overall incidence, patient-level analyses suggest that AEs tend to cluster in susceptible individuals rather than being driven by formulation switching. Gastrointestinal events, the most commonly reported in our cohort, showed directionally consistent associations with baseline comorbidities across both treatment periods, supporting the concept that tolerability is largely modulated by patient-specific factors. This interpretation is consistent with pharmacokinetic evidence indicating that pirfenidone-related gastrointestinal toxicity is primarily influenced by peak plasma concentrations and dosing conditions, rather than by differences in formulation or total drug exposure [26]. Post-marketing surveillance and pharmacovigilance analyses further support the absence of clinically meaningful safety differences between pirfenidone formulations. Large database studies have shown similar reporting patterns for pirfenidone-related AEs, with serious reactions—such as drug-induced liver injury or severe cutaneous adverse reactions—remaining rare and not formulation-specific [16, 27]. In this context, the absence of increased AEs or treatment discontinuations following formulation switching in our study reasonably provides reassurance regarding the tolerability of generic pirfenidone in routine clinical practice. However, due to the relatively small number of events and the limited follow-up period, our study is not powered to evaluate rare or delayed AE and definitive conclusions regarding safety equivalence remain to be further investigated.

### Limitations

Several limitations should be acknowledged. First, this was a single-centre retrospective study with a relatively limited sample size, and no formal a priori sample size calculation was performed for the equivalence analysis. This study may therefore have been underpowered to exclude small but clinically relevant differences between formulations, and a type II error cannot be ruled out. However, because the switch was policy driven and applied to all eligible patients without physician- or patient-driven preselection, the risk of selection bias was likely reduced. Second, the analysis focused primarily on functional outcomes derived from spirometry and diffusing capacity, without systematic assessment of other relevant physiological domains, such as total lung capacity, or the inclusion of absolute FVC values in millilitres, which may have provided complementary information on disease progression. Third, although the within-patient design reduces interindividual variability, the before-after comparison remains susceptible to temporal influences such as natural disease progression, regression to the mean, and time-dependent confounding. In IPF, short-term changes in lung function may occur independently of treatment formulation because of the inherent variability of disease trajectories. Moreover, AEs were collected through routine clinical documentation and patient self-reporting rather than through a structured adjudication process, potentially leading to underreporting or misclassification; however, this approach reflects real-world clinical practice and captures tolerability as experienced by patients outside controlled trial settings. Finally, adherence, dose adjustments, and short treatment interruptions were not quantified in detail. However, all patients had been receiving branded pirfenidone for at least 6 months before switching, implying that baseline tolerability and dose stabilization had already been established. As a consequence, due to the specific study design, early treatment discontinuation due to rapid disease progression or pirfenidone intolerance in general was not represented in this cohort. Residual confounding inherent to observational designs cannot be fully excluded.

## Conclusions

In this real-world, within-patient study of patients with IPF, switching from branded to generic pirfenidone was not associated with clinically meaningful differences in lung function decline or treatment tolerability over a 6-month period. At the patient level, the incidence and pattern of AEs were comparable between branded and generic formulations, with no signal of formulation-specific toxicity or increased treatment discontinuation. These findings are consistent with the known pharmacological profile of pirfenidone and suggest that tolerability is largely driven by patient-related factors rather than formulation differences. Taken together, the present data provide clinically relevant reassurance that policy-driven switching from branded to generic pirfenidone does not appear to alter short-term functional outcomes or safety in routine clinical practice. Further prospective and multicentre studies are warranted to confirm these findings over longer follow-up periods and across broader IPF populations.

## Supplementary Information

Below is the link to the electronic supplementary material.

Supplementary file1 (DOCX 31 KB)

## Data Availability

Data are available upon motivated request at the Respiratory Disease Unit of the University Hospital of Modena, Italy.

## Declarations

### Generative AI and AI-assisted technologies in the writing process

During the preparation of this work the authors used ChatGPT in order to improve language and readability. After using this tool/service, the authors reviewed and edited the content as needed and take full responsibility for the content of the publication.
