# Supplementary material for: Efficacy and tolerability of generic pirfenidone after switch from Esbriet® in idiopathic pulmonary fibrosis: a real-world observational study
Source: Intern Emerg Med. 2026 Apr 17;21(5):1625–33. doi: 10.1007/s11739-026-04343-9 (PMC13452846; doi:10.1007/s11739-026-04343-9)
Supplement: Supplementary file 1 [file 11739_2026_4343_MOESM1_ESM.docx]

# Supplementary Materials.

# eTable 1. Detailed event-level distribution of treatment-related adverse events

| Adverse event category | Specific adverse event | Total events (n) | Branded pirfenidone (n) | Generic pirfenidone (n) | Severity (CTCAE v4.0) |
| --- | --- | --- | --- | --- | --- |
| Gastrointestinal AE | |  |  |  |  |
|  | Nausea | 8 | 4 | 4 | Mild–Moderate |
|  | Dyspepsia | 9 | 4 | 5 | Mild–Moderate |
|  | Vomiting | 4 | 0 | 4 | Mild–Moderate |
|  | Diarrhea | 4 | 1 | 3 | Mild–Moderate |
|  | Abdominal distension | 2 | 0 | 2 | Moderate |
|  | Gastric obstruction | 1 | 0 | 1 | Moderate |
| Skin-related AE |  |  |  |  |  |
|  | Photosensitivity reaction | 6 | 4 | 2 | Mild–Moderate |
|  | Rash | 1 | 0 | 1 | Moderate |
|  | Pruritus | 1 | 1 | 0 | Mild |
| General / systemic AE | |  |  |  |  |
|  | Weight loss | 8 | 4 | 4 | Moderate |
|  | Asthenia | 3 | 3 | 0 | Moderate |
|  | Sleepiness | 2 | 1 | 1 | Mild–Moderate |
|  | Decreased appetite | 3 | 2 | 1 | Moderate |
| Other AE |  |  |  |  |  |
|  | Arthralgia | 1 | 0 | 1 | Moderate |
|  | Headache | 1 | 0 | 1 | Moderate |
|  | Anemia | 1 | 0 | 1 | Mild |
|  | Confusion | 1 | 1 | 0 | Mild |
|  | Increased GGT | 1 | 1 | 0 | Moderate |
|  | Acute kidney injury | 1 | 1 | 0 | Moderate |

**eTable 1**. This table reports the detailed, event-level distribution of treatment-related adverse events observed during therapy with branded and generic pirfenidone. Adverse events are classified by category and specific event, with the number of occurrences reported separately for each treatment period. Event severity is graded according to the Common Terminology Criteria for Adverse Events (CTCAE), version 4.0. Multiple adverse events could occur in the same patient.

*Abbreviations*

AE, adverse event; CTCAE, Common Terminology Criteria for Adverse Events; GGT, gamma-glutamyl transferase.
